# Prevalence and severity of physical intimate partner violence during pregnancy among adolescents in eight sub-Saharan Africa countries: A cross-sectional study

**DOI:** 10.1371/journal.pgph.0002638

**Published:** 2024-07-16

**Authors:** Caroline Adjimi Nyemgah, Meghna Ranganathan, Doreen Nabukalu, Heidi Stöckl

**Affiliations:** 1 Department of Global Health and Development, Faculty of Public Health and Policy, London School of Hygiene and Tropical Medicine, London, United Kingdom; 2 Department of Statistics, Faculty of Epidemiology and Population Health, London School of Hygiene and Tropical Medicine, London, United Kingdom; 3 Institute of Medical Information Processing, Biometry and Epidemiology, Public Health and Health Services Research, Faculty of Medicine, LMU Munich, Munich, Germany; 4 Pettenkofer School of Public Health, Munich, Germany; The University of Newcastle Australia: University of Newcastle, AUSTRALIA

## Abstract

Globally, intimate partner violence (IPV) is highly prevalent, with adolescents being particularly vulnerable, especially during pregnancy. This study examines the prevalence and severity of physical IPV among pregnant adolescents in sub-Saharan Africa (SSA). We analyzed data from Demographic Health Surveys collected between 2017–2021 from eight SSA countries, involving 2,289 ever-pregnant adolescents aged 15–19. Physical IPV during pregnancy was defined as experiencing physical harm while pregnant by a husband, former partner, current boyfriend, or former boyfriend. Severity of physical IPV included experiences such as kicking, choking, weapon threats, and serious injuries. Logistic regression analysis was conducted, with results presented as unadjusted and adjusted odds ratios with 95% confidence intervals. The prevalence of physical IPV during pregnancy among adolescents in the eight SSA countries ranged from 2.9% to 12.6%, with 5.6% experiencing severe lifetime physical IPV and 6.3% severe physical injuries. We found a strong association between physical IPV during pregnancy and severe lifetime physical IPV (aOR: 6.8, 95% CI: 4.5–10.4) and severe injuries (aOR: 9.2, 95% CI: 6.0–14.2), even after adjusting for covariates. Physical IPV during pregnancy is common among adolescents in SSA and is associated with severe physical lifetime IPV. Addressing this issue in low-resource settings requires collaborative efforts among community stakeholders, health system practitioners, and policymakers to protect vulnerable adolescent girls during pregnancy.

## Introduction

Intimate partner violence (IPV) is defined as physical, sexual, or psychological abuse by a current or former spouse or partner and is known to often result in serious physical and psychosocial consequences [1]. The ramifications of IPV extend beyond the individual level, as it impacts communities and hinders progress toward gender equality, particularly during times when women are most vulnerable, such as pregnancy [2, 3]. The prevalence of IPV varies significantly across countries and settings, emphasising the complex interplay of cultural, social, and economic factors [4, 5]. In the context of reproductive health, the World Health Organization’s (WHO) multi-country study on IPV highlighted a wide-ranging prevalence of IPV, with estimates varying from 1.2% to 66%, and a global average prevalence of 30% [6–8]. Among Women of reproductive age (15–49), 27% reported experiencing physical or sexual IPV in their lifetime, emphasising the pervasive nature of the problem [9]. Among women of reproductive age, adolescents and young women face heightened vulnerability, with research consistently demonstrating their elevated risk of experiencing IPV. Adolescent girls and young women (15–24 years old) have the greatest prevalence of recent IPV [9]. Despite its significance, the prevalence and severity of IPV during pregnancy among adolescents in SSA remains understudied and inconsistently reported.

A multi-country, cross-sectional survey conducted in urban areas of five countries, including Baltimore, Delhi, Ibadan, Johannesburg, and Shanghai, highlights the prevalence associated with the experience of IPV. The findings revealed that Johannesburg in South Africa scored the highest prevalence of IPV (36.6%), with pregnancy being one of the leading contributing factors for increasing IPV [4]. A comprehensive analysis of IPV prevalence during pregnancy across 19 countries was conducted by Devries et al. using data from the Demographic and Health Survey (DHS) and the International Violence Against Women Surveys (IVAWS) collected between 1998 and 2007. Their results revealed that IPV during pregnancy is alarmingly common. Prevalence rates ranged from approximately 2.0% in some countries like Australia, Cambodia, Denmark, and the Philippines, to as high as 13.5% in Uganda among ever-pregnant, ever-partnered women. Notably, prevalence tended to be higher in African and Latin American countries compared to European and Asian nations surveyed, with half of the surveys estimating prevalence rates between 3.9% and 8.7% [8]. A systematic review of various forms of IPV during pregnancy in antenatal care in SSA revealed overall rates ranging from 2% to 57%, with physical violence experienced by 22.5% to 40% of cases [10]. Another research conducted in antenatal care at a national referral hospital in Dar es Salaam on the prevalence of physical and/or sexual assault in the index pregnancy showed 27% [11].

The detrimental effects of IPV during pregnancy on maternal and child health are well-documented, with studies linking IPV to adverse outcomes such as low birth weight, preterm delivery, and postpartum depression [12]. One study in Cameroon involving adolescents has linked adverse health outcomes during delivery with IPV during pregnancy [13]. The study analysed maternity reports from 2009–2016 to determine delivery outcomes between primiparous and multiparous adolescents in two primary healthcare facilities that covered most of Cameroon’s rural area deliveries. The analysis revealed that the primiparous adolescents were three times more likely to have low birth weight infants and a history of physical, sexual, and psychological violence during pregnancy [13]. Another study examining the impact of IPV during pregnancy on 61 adolescents postpartum in South Africa showed that 40% of adolescents reported experiencing physical IPV during pregnancy [14]. More importantly, adolescents who reported IPV during pregnancy were also more likely to be infected by a sexually transmitted disease (STD) at six months postpartum than those who did not report it [14]. Another study from Durban, South Africa, investigating IPV and its effect on the mental health of pregnant adolescents between the ages of 14 to 21, found lower odds of having depression, anxiety, and prenatal distress among adolescents who did not report IPV during pregnancy [20]. However, adolescents who reported experiencing IPV experienced all three outcomes, including depression, anxiety, and prenatal distress [15]. Qualitative research conducted in Eastern Kenya highlighted the link between experiencing physical IPV and suicidal ideation risk among pregnant adolescents using 27 focus group discussions, emphasizing the urgent need for targeted interventions [16].

Pregnant women may also face additional stressors like stigma, discrimination, and lack of support in the context of SSA countries, where deeply ingrained cultural and social norms intersect with violent dynamics that increase their likelihood of experiencing IPV [17]. Evidence suggests that pregnancy serves as a unique period marked by increased stress levels, heightened physical susceptibility, and shifting dynamics within intimate relationships, all of which contribute to a heightened risk of IPV [16, 18, 19]. Adolescents, particularly pregnant women, are at a higher risk of experiencing IPV due to limited autonomy, financial resources, and decision-making power [4]. The occurrence of violence during pregnancy is also influenced by factors that also trigger IPV outside pregnancy such as place of residence, education level, marital status, socioeconomic status (SES), and employment status [20–24]. For instance, place of residence may reflect differential access to resources and support systems, with urban areas potentially offering more avenues for seeking help and support services. Education level is often linked to empowerment and awareness, potentially influencing women’s ability to recognize and report IPV. Marital status can affect women’s vulnerability to IPV, with factors such as cohabitation status and relationship dynamics playing a role. Socio-economic status captures broader socioeconomic disparities that could impact access to healthcare and support services. Additionally, employment status may influence women’s economic independence and ability to leave abusive relationships.

Despite the strong evidence on the prevalence and health outcomes of IPV during pregnancy, our study is the first to our knowledge to investigate the link between IPV during pregnancy and severe lifetime IPV experiences and injuries due to IPV among adolescents in SSA using nationally representative data. While the selected outcome variables aim to measure the severity of physical IPV, it is essential to acknowledge that the association between physical injuries and physical IPV during pregnancy might seem intuitively strong. There is no existing evidence for it, hence we believe it is crucial to provide empirical proof. Providing evidence of this link will allow us to put more pressure on calls for creating tailored interventions for adolescents, as it is further proof of the impact of physical IPV during pregnancy on adolescent women’s health.

## Methods

### Study design and setting

We used the population-based household DHS data collected between 2017 and 2021 in SSA. The DHS runs every five to six years across various countries, primarily in low and middle-income countries (LMICs). A standardized set of questions on domestic violence (DV) was created in late 1990 to be optionally added to the DHS, including also capturing data on the frequency of physical IPV during pregnancy [8]. Census enumerator regions are chosen in the first stage of a multi-stage sample selection process with probability proportionate to size. From a comprehensive list of households within the selected enumerator zones, households were randomly chosen.

### Study population and recruitment

**Fig 1:**
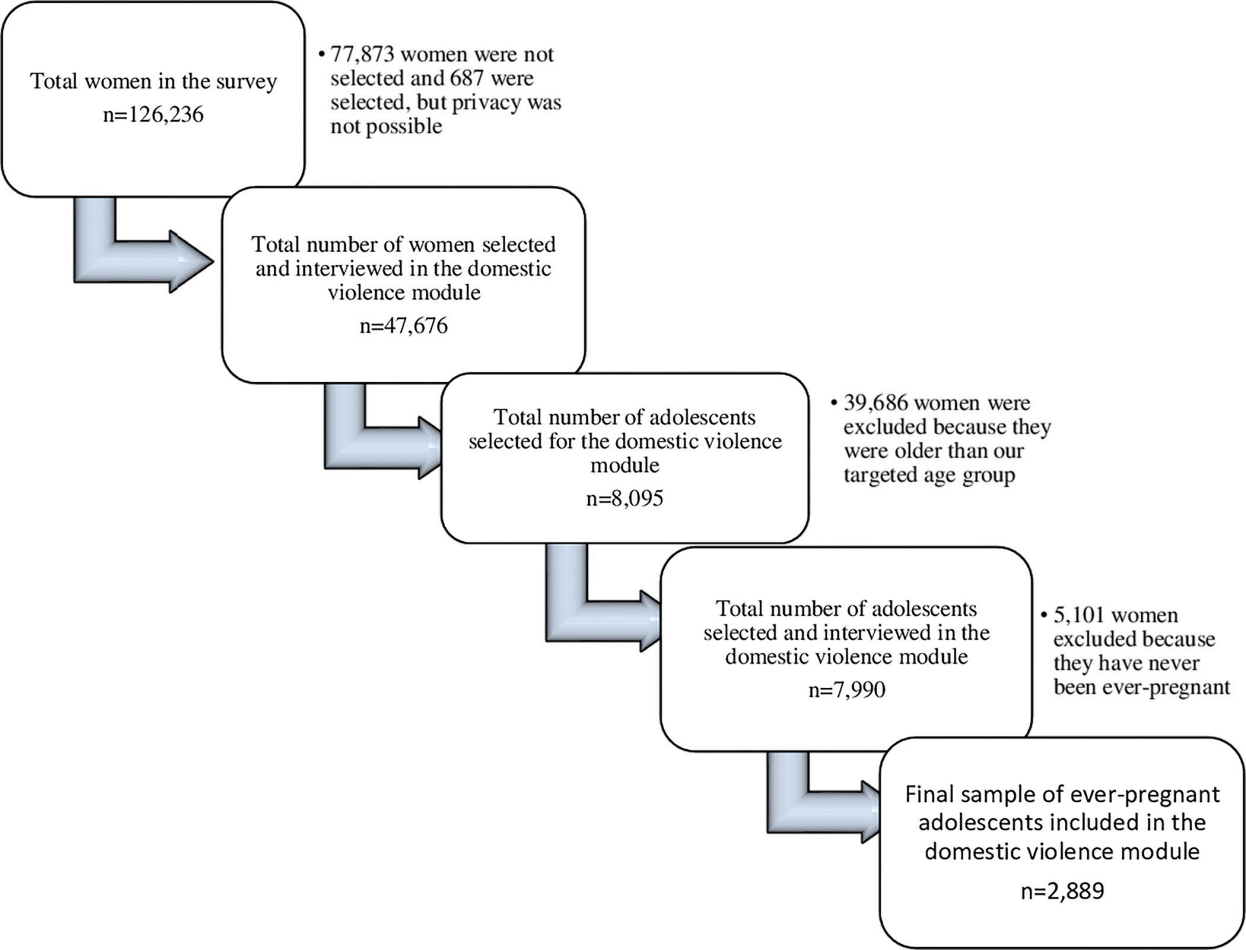
Selection procedure of the study sample from the DHS in eight countries.

This sample included a nationally representative sample of 126,236 women between 15 to 49 years of age selected for this study, capturing 47,676 women who completed the DV module, with 8,095 adolescents in the sample (See Table 1). As the current study is focused on adolescents’ pregnancy, the sample was further restricted to 2289 ever-pregnant adolescents (ages 15–19) who completed the DV module (Fig 1).

**Table 1:** Sample of all ever-pregnant adolescents selected and interviewed for the domestic violence module.

Sample of all ever pregnant women by country	Survey years	Number of women sampled in the DHS	Number of women selected and included in the DV module	Number of adolescents included in the DV module	Number of ever-pregnant adolescents included in the DV module
**Burundi**	2017	17,269	10,188	1,787	197
**Cameroon**	2018	10,656	3,290	690	229
**Liberia**	2019	8,065	3,120	533	235
**Mali**	2018	10,519	3,784	650	298
**Nigeria**	2018	41,821	10,678	1,557	437
**Senegal**	2019	15,574	5,248	866	239
**Sierra Leone**	2019	8,649	1,865	365	67
**Zambia**	2018	13,683	9,503	1,647	587
**Total**		**126,236**	**47,676**	**8,095**	**2,289**

To ensure confidentiality and safety, a statement requesting informed permission is was read to the respondent before each interview that they were free to accept or decline. Before a child or adolescent can participate, their parent or guardian must provide their consent. The DHS DV module was completed by one randomly eligible woman in every third household using the WHO violence against women ethical guidelines for conducting DV research [8]. Interviewers were recruited and specially trained to administer the DV module. The training includes ensuring confidentiality, privacy and building rapport with the respondent [8]. A written authorization letter was obtained from the DHS Institutional Research Board (IRB) for public usage and ethical approval was received from the London School of Hygiene and Tropical Medicine (LSHTM) for this secondary data analysis.

### Variables and measurement

#### Explanatory variable

*Physical IPV during pregnancy*. The main explanatory variable for this study was physical IPV during pregnancy This variable represents whether a woman has experienced physical abuse from an intimate partner including a husband, former partner, current boyfriend, or former boyfriend while pregnant. It is assessed through a question asking ‘if anyone has hit, slapped, kicked, or done anything else to hurt the woman while she was pregnant. The only response option given in the DHS is binary (yes/no)’. An affirmative answer to the question constituted physical IPV during pregnancy (Table 2).

**Table 2:** Demographic and health survey variable definitions.

Sample	Definition of perpetrators	Form of violence	Timeline	Violence question	
Ever pregnant woman 15–19 capturing current pregnancies that ended in stillbirth, miscarriages, or induced abortions.	Husband, spouse, former partner, current boyfriend, and former boyfriend	Physical violence	During pregnancy and lifetime	During pregnancy	Lifetime severe physical violence
				Has anyone ever hit, slapped, kicked, or done anything else to hurt you physically?	Ever had bruises because of husband/partner’s actions; ever been "kicked or dragged; tried to strangle or burn; threatened with knife/gun or other weapons because of husband/partner.

#### Outcome variables

To determine the severity of physical IPV, we used the WHO classification of severity of violence [20] to capture two variables in the DHS.

*Lifetime severe physical injuries*. This variable captures whether a woman has experienced severe physical injuries as a result of IPV at any point in her life. It is measured based on responses to questions regarding specific types of injuries such as eye injuries, sprains, dislocations, burns, deep wounds, broken bones, and other serious injuries resulting from IPV.

*Severe physical IPV in the last 12 months*. This variable indicates whether a woman has experienced severe physical IPV, including being kicked or dragged, strangled or burned, or threatened with a knife/gun or other weapons by a husband or any other current or previous male partner in the last 12 months preceding the survey. It is also a binary variable (yes/no).

#### Covariates

Based on our literature review [20–22] and conceptual thinking, the following five variables were chosen as covariates: place of residence, education level, marital status, socio-economic status (SES), and employment status. The variable, place of residence categorises respondents based on whether they live in urban or rural areas. The education level captures no education, primary, secondary, or higher education. Marital status classifies respondents based on whether they were never in union, currently in union/living with a man, or formerly in union. The SES variable, following the given DHS coding, classifies respondents into different socio-economic groups, such as poorest, poorer, middle, richer, or richest, while employment status indicates whether respondents are employed or not employed.

#### Data management and analysis

We used Stata version 16 SE for the analyses. The prevalence calculation was done separately for each country and merged. One woman is selected in each household, and each survey is weighted to adjust for non-response. Following the recommendation from the DHS and the survey command, the women’s sample weights for the DV module (d005/1,000,000) were used to produce unbiased estimates. The prevalence was extracted using inferential statistics in adolescents, including percentages, and illustrated using charts of adolescents aged 15–19 years (see Table 3).

**Table 3:** Characteristics of adolescents included in this study.

Place of residence	*n*	*%*
Urban	606	26.4
Rural	1683	73.5
**Level of education**		
No education	685	29.9
Primary	866	37.8
Secondary	737	32.2
Higher	1	0.0
**Marital status**		
Never in union	620	27.0
Currently in union/living with a man	1605	70.1
	64	2.8
Formerly in union		
**Employment status**		
Not employed	1188	51.9
Employed	1101	48.1
**Socio-economic status**		
Poorest	643	28.0
Poorer	592	25.8
Middle	491	21.4
Richer	369	16.1
Richest	194	8.4

The chi-square test of independence was used to determine the associations between physical IPV during pregnancy, lifetime severe physical IPV, and severe injuries in each of the eight countries. Two logistic regressions were run to examine the association between physical IPV during pregnancy, lifetime severe physical IPV, and severe IPV-related injuries. The results were presented using odd ratios with 95% confidence intervals and significance estimated with a p-value < 0.001.

## Results

### Characteristics of study participants

A total of 2,289 adolescents were included in the current study. The majority of adolescents lived in rural areas (73.5%), had a primary level education (37.8%), and were currently living with a man (70.1%). A little more than half were not employed (51.9%), with 28.09% being among the poorest adolescents (Table 2). Out of 2,289 adolescents selected and interviewed in the DV module, 179 (Table 3) had reported physical IPV during pregnancy from which 45 (25,1%) have also reported severe physical injuries due to violence by a partner, and 43 (24%) have reported experiencing lifetime severe physical IPV.

### Prevalence of physical IPV during pregnancy

**Fig 2:**
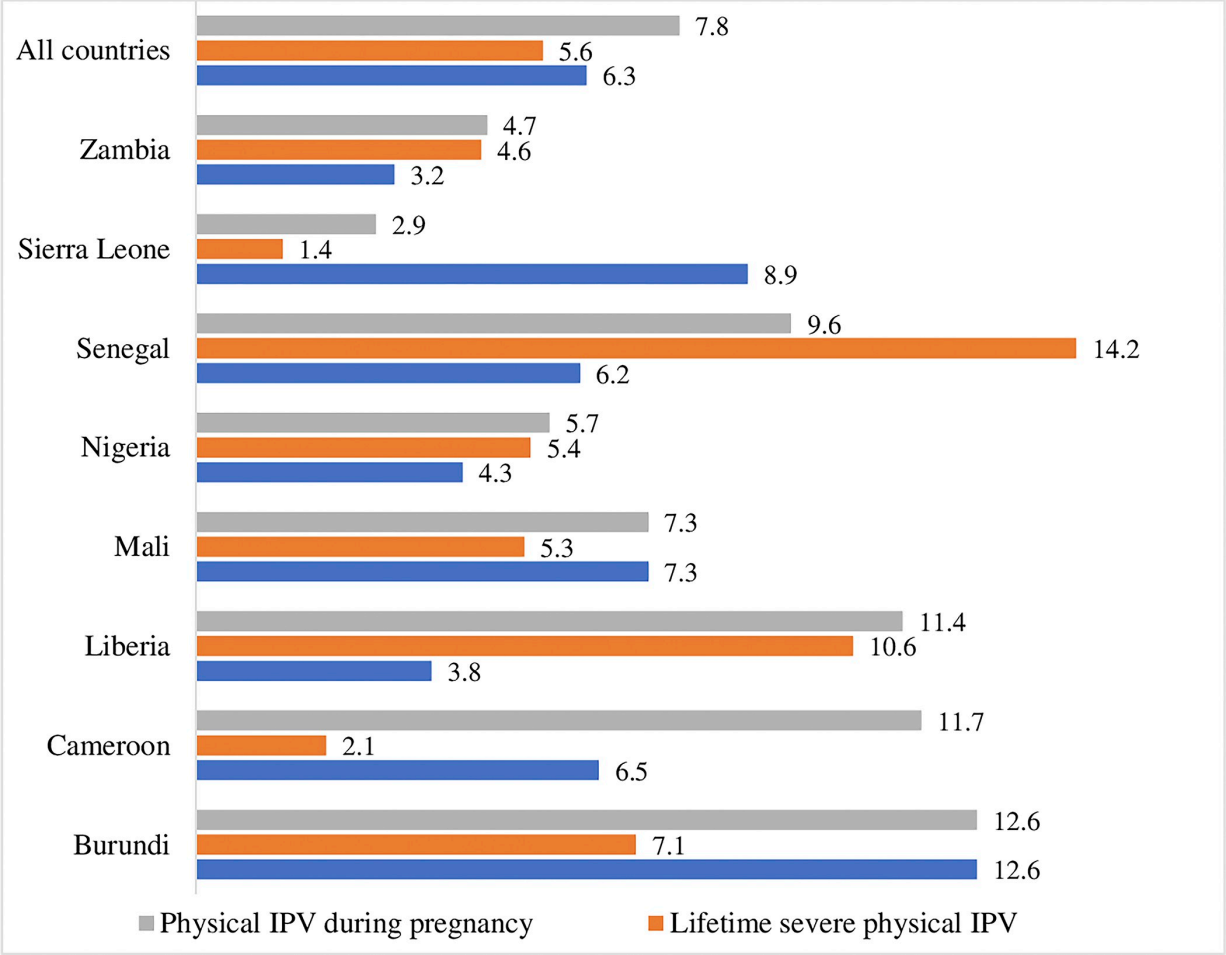
Prevalence of physical IPV during pregnancy, lifetime severe physical IPV, and physical injuries by partner (%) among adolescents by country.

The prevalence of physical IPV during pregnancy among ever-pregnant adolescents across all countries is 7.8%, with the highest prevalence of 12.6% in Burundi and the lowest at 2.9% in Sierra Leone (Fig 2). The lifetime severe physical IPV and physical injuries by partners among ever-pregnant adolescents were 6.3% and 5.6%, respectively. The prevalence of injuries resulting from physical IPV ranges from 1.4% in Sierra Leone to 12.6% in Burundi, while reports of severe lifetime physical IPV range from 3.2% in Zambia to 14.2% in Senegal.

### Association between lifetime severe physical IPV and physical injuries by partners among ever-pregnant adolescents who reported physical IPV during pregnancy

**Fig 3:**
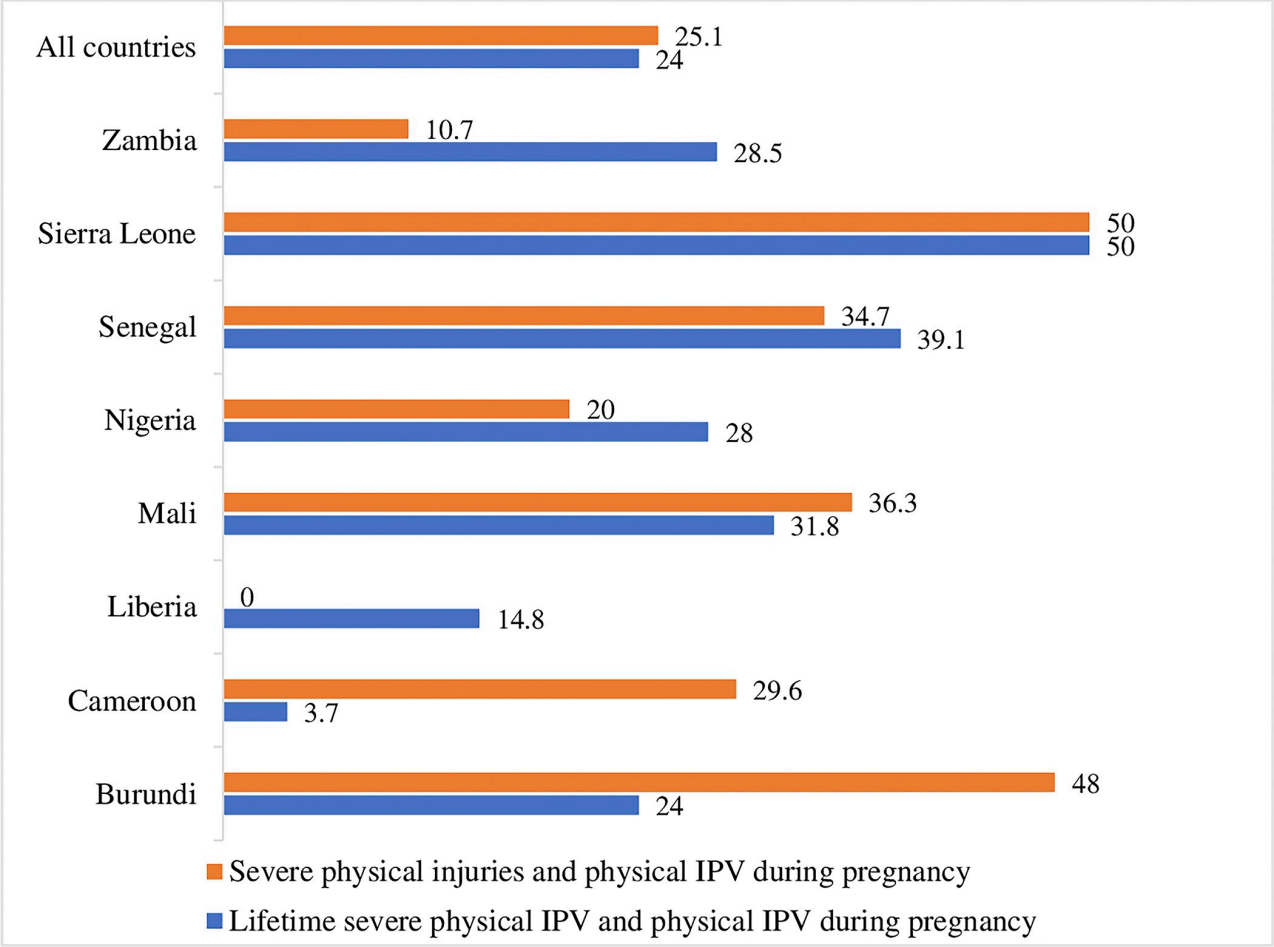
Prevalence associations between lifetime severe physical IPV and physical injuries by partners among ever-pregnant adolescents reporting physical IPV during pregnancy.

Table 4 below displays the sample sizes of included adolescents, the prevalence of IPV during pregnancy, and a p-value to show that the strength of association with lifetime severe physical IPV and physical injuries by partners among ever-pregnant adolescents who reported physical IPV during pregnancy. Fig 3 visualises these findings. In all eight countries, the relationship between physical IPV during pregnancy, severe lifetime physical IPV, and physical injuries by a partner is significant among adolescents in six countries. No association was found with severe lifetime physical IPV in Cameroon and Liberia. In Liberia, no association was found with severe physical injuries due to violence by partners. It is important to point out that these results are probably due to the low number of participants observed in the sample size.

**Table 4:** Association between lifetime severe physical IPV and severe physical injuries by partner among ever-pregnant adolescents reporting physical IPV during pregnancy.

Country	Adolescent in domestic violence module	Number of adolescents who reported physical IPV during pregnancy	Lifetime severe physical IPV and physical IPV during pregnancy			Severe physical injuries and physical IPV during pregnancy		
			Count	%	P-value	Count	%	P-value
**Burundi**	197	25	6	24%	0.000	12	48	0.000
**Cameroun**	229	27	1	3.7	0.5	8	29.6	0.000
**Liberia**	235	27	4	14.8	0.4	0	0	0.2
**Mali**	298	22	7	31.8	0.000	8	36.3	0.000
**Nigeria**	437	25	7	28	0.000	5	20	0.000
**Senegal**	239	23	9	39.1	0.000	8	34.7	0.000
**Sierra Leone**	67	2	1	50	0.000	1	50	0.03
**Zambia**	587	28	8	28.5	0.000	3	10.7	0.02
**All countries**	2289	179	43	24.0	0.000	45	25.1	0.000

The results of the logistic regression analysis for the combined eight countries included in this study showed that the the odds of physical IPV were higher among adolescents who have experienced physical injuries due to IPV (OR = 10.5, 95% CI = 9.71–11.4) and lifetime severe physical IPV (OR = 8.1, 95% CI = 7.5–8.8) even after controlling for covariates; for both lifetime severe physical IPV (aOR = 9.2, 6.0–14.2) and physical injuries (aOR = 6.8, 4.4–10.4) (Table 5).

**Table 5:** Multivariable models showing the association between physical IPV during pregnancy with physical injuries and lifetime severe physical IPV.

Model 1: Association of physical IPV during pregnancy and physical injuries			Model 2: Association of physical IPV during pregnancy and lifetime severe physical violence	
Countries	COR [95% CI]	AOR [95% CI]	COR [95% CI]	AOR [95% CI]
**Burundi**	100,6 [39.8–254.1] ***	7.9 [2.7–23.3] ***	61.4 [19.9–189.8] ***	3.8 [1.0–13.9] *
**Cameroun**	38.1 [12.7–118.1] ***	15.9 [4.5–55.2] ***	6.2 [0.6–57.8]	1.8 [0.1–20.4]
**Liberia**	0.0 [.01–0.0] ***	Low observations	3.3 [1.0–10.2]*	2.8 [0.6–12.1]
**Mali**	20 [7.4–54.1] ***	21.1 [6.1–72.9] ***	23.3 [8.0–67.7] ***	18.2 [5.6–58.9] ***
**Nigeria**	21.8 [7.3–65.0] ***	10.3 [3.2–32.9] ***	26.2 [9.9–69] ***	11.5 [4.0–33.0] ***
**Senegal**	48.8 [16.6–144] ***	22.2 [6.1–80.2] ***	15.1 [6.1–37.4] ***	6.1 [1.9–19.4] ***
**Sierra Leone**	71.4 [3.9–1309] ***	Low observations	356 [12.0–10,777] ***	Low observations
**Zambia**	10.4 [2.8–37.7] ***	4.9 [1.1–21.2] ***	28.4 [11.3–71.3] ***	17.7 [5.5–56.8] ***
**All countries**	25.6 [17.3–37.8] ***	9.2 [6.0–14.2] ***	8.1 [7.5–8.9] ***	6.8 [4.4–10.4] ***

**Model 1:** unadjusted and adjusted model examining the independent association of physical IPV during pregnancy and physical injuries

**Model 2:** unadjusted and adjusted model examining the independent association of physical IPV during pregnancy and lifetime severe physical violence

*p < 0.05

**p < 0.01

***p < 0.001.

## Discussion

The study provides valuable insights into the prevalence and potential implications of physical IPV during pregnancy among adolescents in SSA. It is worth noting that this analysis is the first to examine this issue in SSA using the DHS, making its findings significant for both the region and the broader understanding of IPV during pregnancy. The study identified that 7.8% of pregnant adolescents in the eight SSA countries disclosed experiencing physical IPV during their pregnancies, out of which 6.3% reported severe lifetime physical IPV and 5.6% partner-inflicted physical injuries. Our data suggests that physical IPV during pregnancy is prevalent among adolescents in SSA, aligning with previous results by the Devries et al (2010) multi-country analysis of IPV prevalence during pregnancy from 19 countries [8]. However, it can be challenging to identify the true prevalence of physical IPV during pregnancy for various reasons, including fear of retaliation if the offender were to learn about it through disclosure or any guilt felt by the woman exposed to IPV [25] and hence non-response bias. Similar research on pregnant adults has identified a tendency to underestimate IPV prevalence during early pregnancy due to this bias [26].

Although our results are lower than one found in a study in eastern Ethiopia that demonstrated that pregnancy could offer protection during pregnancy [21], the results from our analysis indicate that physical IPV during pregnancy is likely to be a marker for severe physical IPV beyond adolescent pregnancy with physical IPV during pregnancy not being an isolated event but part of a broader pattern of abuse. Moreover, countries reporting a high percentage of severe lifetime physical IPV and injuries due to violence by a partner also reported high levels of physical IPV during pregnancy, except for Liberia and Cameroon. In this study, more than 10% of adolescents in each country who reported physical IPV during pregnancy reported severe IPV including physical injuries and severe lifetime physical IPV. Earlier research from both SSA and other regions have provided context for this finding, namely that IPV during pregnancy can increase in frequency and severity [26, 27], as well as in Europe in a study of adult women receiving antenatal care in six northern European countries [28]. Our findings suggest that adolescents could be as likely as adults and even experience more severe physical IPV. Therefore, physical IPV during pregnancy may not only be a continuation of prior IPV but also a marker for severe physical violence [29]. Potential reasons for this are the partner’s sexual frustration, the stress of expecting a child, the heightened physical vulnerability of the pregnant woman, and a conscious or unconscious wish to end the pregnancy [12].

It is essential to consider that cultural and contextual factors could contribute to variations in prevalence across countries, potentially leading adolescents to perceive and accept IPV as normal within societies where there is a higher tolerance for such violence [30]. It is also crucial to acknowledge the possibility that some women who have experienced IPV may suppress their distress as a coping mechanism, which could deter them from reporting their experiences [27]. Adolescents may normalise their experiences of IPV in a society with a higher tolerance for IPV. It is also possible that adolescents included in the current study have recovered from previous physical IPV, may have experienced one instance of physical IPV only or a minor form of it, or have higher levels of resilience. Nevertheless, we cannot rule out the possibility that women who have experienced IPV may repress their distress as a coping mechanism, that may prevent them from reporting [27]. This finding also implies that adolescents who experience IPV during pregnancy are more likely to have a history of severe violence and may continue to face severe IPV or IPV-related injuries in the future [8].

It is essential to acknowledge prior studies that contribute to the broader understanding of IPV among adolescents in different contexts, including SSA. These studies provide valuable insights into the complex dynamics of IPV during adolescence and its potential health implications. The multi-country cross-sectional survey conducted in urban areas of Baltimore, Delhi, Ibadan, Johannesburg, and Shanghai revealed important findings regarding the prevalence and contributing factors of IPV among adolescents [4]. Notably, the high prevalence of IPV in Johannesburg, South Africa (36.6%), with pregnancy identified as a leading contributing factor, underscores the significance of the issue. This research highlights the urban setting and provides a basis for comparison with the predominantly SSA-focused analysis in our study. This previous study emphasizes pregnancy as a contributing factor to increased IPV. This aligns with our findings, suggesting that physical IPV during pregnancy among adolescents in SSA could be a marker for severe physical violence. These converging results point out the importance of addressing the unique challenges faced by pregnant adolescents concerning IPV across various settings.

Additionally, exploring physical IPV during pregnancy among adolescents in SSA using the DHS is relevant since it illustrates the potential health consequences including miscarriages, stillbirths, and induced abortions, which is a critical aspect that warrants consideration as Ahinkorah and colleagues have found [31]. The prevalence and impact of IPV can vary significantly between urban and rural settings, as well as across different regions. While the Johannesburg study highlights the high prevalence of IPV in an urban context, our analysis focuses on a broader SSA context, encompassing both urban and rural areas, to provide a comprehensive view of the issue. This distinction is essential as it acknowledges the diverse socio-cultural and economic landscapes within SSA.

### Policy implications and research recommendations

We draw on the findings to emphasise that physical IPV during pregnancy among adolescents in SSA should not be seen in isolation but rather as a dynamic interplay of different factors. Adolescents in SSA frequently encounter particular difficulties, such as their emotional and financial dependence on their male companions. For this reason, pregnancy may aggravate these vulnerabilities, resulting in physical IPV. Our study emphasises the need to tackle gender inequality as a crucial first step in reducing IPV among adolescent mothers in SSA. Jewkes et al. (2002)’s show that persistent gender norms that uphold male dominance and female subordination help to normalise IPV, making it more likely to happen during times like pregnancy [32]. The inclusion of gender equity in interventions and policies empowers girls and questions conventional gender norms. When faced with IPV, especially during pregnancy when vulnerability is increased, these interventions can enable women to stand up for their rights and seek support. In addition to promoting healthier and more equitable relationships and encouraging males to reject violent behaviour, girls’ empowerment will not only improve women’s well-being but also foster a more just and secure environment for both pregnant mothers and their unborn children.

Future research needs to explore men’s perspectives on IPV during pregnancy. Effective intervention and prevention efforts depend on having a complete understanding of the causes and motivations that drive physical IPV during pregnancy. Notably the DHS data primarily consists of information from women who have experienced IPV, therefore future research will tremendously benefit from directly interviewing men who are the perpetrators. Gathering information from male offenders can shed light on the intricate dynamics that result in physical IPV during pregnancy among adolescents. Highlighting the causes of physical IPV during pregnancy, can offer insights into their viewpoints, motivations, and prospective triggers. This strategy would improve our understanding of the issue and help create focused solutions that target these underlying causes. We can create more successful measures to prevent and resolve IPV during pregnancy and eventually promote healthier and safer relationships for women and their unborn children by taking a comprehensive approach that considers both the victim’s and offender’s viewpoints. Finally, Prenatal care has been shown by preliminary research to be a window of opportunity for identifying and supporting physically abused adolescents during pregnancy [33]. Routine or case-based investigations for physical abuse during pregnancy should focus more on providing treatment for younger women. Since antenatal care typically serves as a woman’s main point of contact with the medical community, it provides health services and support throughout her pregnancy, enabling healthcare professionals to establish the relationship required to effectively manage physical IPV [33].

### Strengths and limitations

The DHS is a credible data source that increases the likelihood of generalizability. Nevertheless, despite this strength, several limitations must be considered. We acknowledge the presence of high odds ratios (e.g., 100.6) in the association between physical IPV during pregnancy and physical injuries, and ask to cautiously interpret these findings. It is plausible that the inflated odds ratios may be attributed to the effect of a small sample size of pregnant adolescents who experienced IPV during pregnancy, particularly when disaggregated at the individual country level, suggesting the need for careful consideration in generalizing the findings. Another important limitation that could substantially impact the findings is the underreporting of IPV during pregnancy. The accuracy and completeness of data gathered through surveys or research studies might be severely impacted by factors including fear and stigma, linguistic obstacles, and lack of understanding about IPV during pregnancy. Finally, the DHS only focused on physical IPV during pregnancy without considering other types of violence, including sexual and emotional abuse, that have been shown to have adverse outcomes for both the mother and child [34]. Despite these limitations, cross-sectional studies like this one play a crucial role in generating hypotheses and identifying associations that warrant further investigation. Future research should utilise longitudinal designs to explore the temporal relationships between physical IPV during pregnancy and severity among adolescents in SSA, allowing for a more nuanced understanding of causality and potential pathways of influence.

## Conclusions

This study shows that physical IPV during pregnancy is highly prevalent among adolescents. This is quite alarming for this age group transitioning into adulthood and the special vulnerabilities that they experience due to pregnancy. Therefore, it is essential to develop and implement programs to address gender inequalities and promote sexual and reproductive health, to ensure that adolescents in these contexts are ensured a life free of violence. Policies and programs should also invest in more research to highlight the multiple health consequences of the adolescents’ experience of IPV during pregnancy in SSA, which are crucial for sexual and reproductive health programs to address IPV [16].

## Supporting information

S1 Checklist STROBE statement—checklist of items that should be included in reports of observational studies. (DOCX)
